# Mr. Buchanan's Illustrations of Acoustic Surgery

**Published:** 1825-07-01

**Authors:** 


*Illustrations of Acoustic Surgery.* By Thomas Buchanan, C.M. &c. Surgeon to the Hull Dispensary for Diseases of the Eye and Ear, &c. &c. 8vo. pp. 118, several plates, London, 1825. Price 9s. 6d. boards.

The two master organs of sense, the eye and the ear, have deservedly attracted more attention from surgeons than those of any of the other senses. Of the two organs abovementioned, however, the eye and its diseases have taken the lead—not from the greater importance of sight than of hearing (the *former* is decidedly of less importance than the *latter*) but because the diseases of the eye are more numerous, and they are more within the reach of surgery. What control, for instance, have we over organic affections of those parts of the auditory apparatus which lie within the petrous portion of the temporal bone, compared with the power we possess in regard to diseases of the humours and coats of the eye?—Still, acoustic surgery is an important branch—and we even find that a whole periodical Journal is to be dedicated to that branch alone. This is probably carrying the thing "ultra fines."

Mr. Buchanan's "Guide to Acoustic Surgery" having been favourably received by the heads of the profession, and by the public at large, the author has been induced to bring forward his "Illustrations" of the same subject, with the hope that they will prove of considerable utility to the surgical profession. The work is divided into eight chapters, to which are added a set of formulæ, and a nomenclature of the diseases of the ear. We shall exhibit a short analysis of some of these chapters to our readers.

1. *Injection of the Meatus.* The ear is to be exposed to the rays of the sun—that is, if there be any sun-beams—and the meatus drawn outwards, or stretched so as to bring the membrana tympani distinctly into view. But after sunset, or in dark cloudy weather, it is very difficult to see to the bottom of the ear, and consequently to ascertain the condition of the membrana tympani. On this account, our author constructed an apparatus capable of concentrating the rays of a candle into the meatus, and by that means illuminating the tube and membrana tympani. A plate of this apparatus is given, to which we refer the reader; as no verbal description would suffice. This inspector or illuminator might be employed, our author thinks, with advantage, for inspecting the vagina and os tincæ —or any deep cavities whatever.

5. Passing over three short chapters on probing, and syringing the meatus, and wounds of the auricle, we come to Chap. V. on the mode of extracting extraneous substances and indurated wax from the meatus. The extraneous substances are generally beads, pebbles, or small nuts. When the foreign body is small, the best mode of extraction is by injections of tepid water.

"For this purpose, the point of the syringe ought to be pressed gently against the edge of the meatus, so that it may occupy as little of the diameter of the tube as possible, and when the injection arrives at the membrana tympani the counter current will be formed there, which will force the bead or substance outwards. If the substance be rather large, it may perhaps remain at the entrance of the meatus, whence it ought to be extracted by means of a pair of forceps." 40.

Some aurists recommend that beads should be extracted by the forceps; but Mr. Buchanan has succeeded by means of tepid injections *alone*, "sometimes after other surgeons had failed, when using both forceps and probe." He recommends a pair of slender and well-tempered forceps, with teeth on the inside of the points, for extracting extraneous substances of wood, &c. when lodged high up in the tube, and near the membrana tympani. When insects, worms, or vermin, lodge in the meatus, "the best mode of extraction is by means of an injection of the infusion of tobacco, which will speedily destroy them, and allay the irritation in the tube." The parts ought to be washed immediately afterwards with an injection of tepid water, and a few drops of oil of almonds, poured into the tube, to prevent the patient catching cold after the warm injection.

"When the meatus is filled with wax, the patient complains of diminution of hearing,—sometimes deafness,—various noises in the ear affected,—particularly when masticating.

"The patient sometimes complains, that after yawning, or some other expansion of the jaws, he hears a sudden noise in the ear affected, similar to a 'crack,' and that immediately afterwards his hearing is restored, but that the noises still continue. On inspecting the ear, the interior parts of the meatus will be found occupied by a large mass of wax, which blocks up the tube and obscures the membrana tympani, except at the superior part, where the wax is detached from the tube, leaving in general a horizontal gap. It is through this aperture that the undulations of sound reach the membrana tympani, and thence the vibrations are communicated to the nervous fasciculi.

"The hearing becomes in a little time, however, gradually more indistinct than formerly; great diminution of hearing, and, shortly afterwards, deafness itself, are the never-failing consequences of inattention to the above symptoms.

"On inspecting the parts, the nature of the complaint may be easily ascertained, by the appearance of a large mass of wax, which blocks up the tube, excludes the view, and impedes the vibrations of the membrana tympani.

"I have frequently found that patients affected with this complaint, had been in the habit of stopping up the mouth of the tube with cotton or wool, which, by some means or other, had been forced inwards, and thus formed a nucleus to the indurated cerumen.

"Surgeons are sometimes in the habit of extracting wax from the meatus by means of the end of a silver needle or probe; a practice which is certainly reprehensible, because of the danger which may be incurred to the membrana tympani by any sudden motion of the head of the patient.

"The following method of extraction will be found to be fully more expeditious, and far more safe, than that of rashly groping in the tube with pointed instruments of any kind.

"The ear complained of should be inspected, and if it appears to be partially filled with wax, a syringful of tepid water, of about 90° Fahrenheit, should be thrown into the meatus, and the thumb clapped on the mouth of the tube immediately after the point of the syringe is withdrawn. The injection ought to be kept in the meatus about three or four minutes.

"The auricle is then to be wrought backwards and forwards, and sometimes elongated, and, by these means, the wax will be loosened from the sides of the meatus.

"Another syringful of tepid water should then be thrown inwards, with more force than the preceding; and this latter injection usually issues out more or less tinged with wax, and frequently brings along with it small bits of that substance, tough and hardened.

"In this manner the operator must proceed with the tepid injection, until the meatus be cleared of the whole of the wax, and the sides of the tube, and the membrana tympani be visible on inspection. In some instances the wax acquires a degree of hardness and tenacity, which would scarcely be credited by those who are unacquainted with the diseases of the ear.

"In these cases, the injection though thrown in frequently, yet seldom (for a length of time at least) forces out the chief cause of complaint, which on inspection is found to consist of one solid mass of hardened wax; which, if it should be loosened, or partially reduced by the repeated ablutions, is perhaps only removed from its original position to be fixed in the middle of the tube, where it bids defiance to the streams of tepid water which issue from the meatus. It is therefore sometimes necessary to apply the auricular forceps to extract these hardened masses, when they are near the mouth of the tube." 49.

6. *Deafness from imperfect Secretion of Wax.* The treatment of this complaint requires discrimination and perseverance in the practitioner. Patients with this affection complain of noises—of a dry rustling sound, particularly during mastication—sometimes ringing noises in the head. There is no acute pain in the ear, but often a dull sensation—at times a total remission of all the above symptoms.

"In the 'Synoptical Table of the Diseases of the Human Ear', this complaint forms the *Genus Imperfectum*, which is divided into three species: viz.

"1st—When the wax is deficient in *quantity*.

"2nd—When deficient in *quality*, and

"3rd—When deficient both in *quantity* and *quality*.*

"In the *first species* the secretion of wax is sometimes so extremely small in quantity, that it is only by a full view and careful inspection of the parts that the secretion can be ascertained to exist; and, even then, it is seen merely to tinge slightly the interior parts of the meatus: but, however small the quantity may be, the quality is good. Patients who are affected with this species usually hear tolerably well in clear fine weather, and when the general health is good, but are apt to be affected with the least irregularity of the atmosphere, which, when dull, moist, or cloudy, causes considerable diminution of hearing.

"As the acuteness of hearing is generally restored on the change of the weather, the patient pays little attention to the symptoms, perhaps for a number of years, until the disease becomes so far confirmed that he is sensible of great diminution of hearing, at times approaching to deafness. He is then alarmed, and applies for medical assistance, which if not immediately successful, often causes great uneasiness, which tends to retard the cure.

"In the *second species* the secretion is more plentiful than in the first, but frequently so altered in colour and quality, as to form two varieties, viz.

> "1st—When it is of a whitish colour and thin—similar to a solution of gum acacia.
>
> "2nd—When only a few of the glands secrete the cerumen, so that we often find small patches of insulated wax distributed in the interior parts of the tube, of a rather darker colour than the healthy secretion.

"This variety has a peculiar tendency to harden; and, when the secretory powers of the glands are active, the cerumen forms into distinct lumps, but generally in one large mass.

"When diminution of hearing proceeds from indurated wax, the interior part of the meatus is almost always found blocked up with one large mass; but yet we find that a small quantity of secretion, similar to the second variety, will sometimes considerably diminish the hearing, especially when it lies close to, or upon the membrana tympani.

"In the *third species* the tube is found clean and dry; and if the meatus can be inspected, so as to obtain a full view of the parts, the membrana tympani will appear clear and shining. The meatus is also liable to be affected with scurvy, when the cuticle frequently becomes so dry as to be easily detached in small pieces, similar to the scales of a fish. The integuments are sometimes swollen, and of a reddish blue colour." 60.

In these three species the cerumenous glands are diseased—and the great object is to restore their healthy action. The *first species* admits most readily of cure, viz. by increasing the

---

"* The particular species may always be determined by a careful examination of the meatus."

action of the glands in the meatus, by warmth and stimulating applications. Two drops of the following formula are recommended to be applied to the interior of the tube every night at bed time.

℞. Acid. pyrolig. [acid. acet. fortius.]
Spir. ætheris sulph.
Ol. terebinth. rect. āā part. æqual. misce.

At the same time he recommends a dose of vinum colchici going to bed every night. The state of the bowels are to be carefully attended to.

In the *second species*, the quality of the secretion is to be altered, and often the quantity augmented. The insulated wax should be removed, and the healthy action of the glands excited.

The *third species* sometimes arises, our author thinks, from cold, as bathing when there is some perspiration on the body. The water having free access to the meatus, the vessels of this part are probably stricken torpid, and the power of healthy secretion impaired. Diminution of hearing ultimately follows this torpid state of the tube. Great care must be taken in the treatment of the second and third species, to put the chylopoietic viscera in order. For this purpose our author recommends a bitter aperient, and antacid mixture, composed of infusion of quassia, rhubarb, and magnesia, twice or thrice a day. The patient should be kept, if possible, in a dry and clear air, where there is no marsh effluvium. Proper exercise, so as to increase the action of the skin, is very necessary. The warm bath at bed time—and afterwards a dose of the pulv. ipecac. compos. with two or three grains of calomel. By the way, we think the dose of pulv. ipeca. comp. (20 grains) is rather large for a nightly allowance. Two grains of opium should not be given, under ordinary circumstances, to a patient every night. Our author has known several instances of this third species of deafness caused by exposure to cold when under the influence of mercury for syphilis, and where the complaint resisted every means of cure until the mercurial influence was again excited. The following injection for the ear, in this complaint, is given by Mr. Buchanan :

℞. Acid. pyrolig. ʒij.

Aquæ distillat. ℥vj. injectio.

"When the practitioner wishes to stimulate the parts, the injection ought to be made every time it is applied, and the point of the syringe held at the distance of about five or six inches from the auricle. The fluid should then be forcibly injected into the meatus, and by this means effervescence will take place, which if confined to the tube will produce more beneficial effects than ten times the quantity of the same fluid *poured* into the meatus." 65.

Blisters behind the auricle are sometimes very beneficial.

7. *Polypi in the Meatus.*—This frequently takes place subsequently to inflammation of the meatus, especially where a scrofulous diathesis obtains. The recent polypus consists of a soft, spongy, reddish-coloured substance, lubricated with a thin fluid secreted by the diseased cerumenous glands and the excrescence. In process of time the tumour acquires a considerable degree of tenacity in its structure, and the secretion becomes purulent, the surface of the tumour having ulcerated. The tumour is connected by a peduncle or stalk to the parietes of the tube, or to the edge of the membrana tympani. Sometimes, however, the neck is as large as the body.

"There is only one complaint to which the meatus is liable, that may be mistaken for polypi, and that is, tumefaction of the cerumenous glands.

"These glands are sometimes so much enlarged that the meatus is completely blocked up, and the patient rendered deaf during the continuance of the complaint. When the parts are in this state one or two of the glands are generally more enlarged than the others, which gives them the appearance of a polypus tumour.

"In order to ascertain the nature of a complaint with these appearances, (should inspection fail) the practitioner ought to endeavour to introduce a probe around the enlargement, and if it be polypi the probe will pass freely around the tumour.

"But if he be unable to pass the probe, except on one side of the tumour, or in the centre of the meatus, and the enlargement be dry, extremely tender to the touch, and the surrounding parts participate in the swelling and inflammation, he may then be assured that the disease is not polypi, but tumefaction of the glands and integuments of the tube." 71.

These polypi will sometimes continue for years, without much other inconvenience to the system than deafness and a puriform secretion, especially where much cleanliness is observed; yet it is at all times a formidable disease, as the external parts of the ear are apt to get disorganized. Hence it is an imperious duty on every practitioner to annihilate, if possible, this excrescence as soon as the situation and size of the neck of the polypus can be ascertained.

"The best method of destroying polypi in the meatus, (in my opinion) is, to extract it by the root, by means of forceps, and afterwards touch the parts with an escharotic or stimulating application.

"Some practitioners prefer caustic instead of the forceps; but when the tumour is large and subjected to this mode of operation, the process is tedious, the pain often excruciating; and besides these disagreeable circumstances, the surrounding parts generally suffer from the destructive power of the caustic.

"It is for these reasons that I prefer the mode of extraction by the forceps; and for this purpose I have been in the habit of using a small pair slightly curved, similar to those of plate III, fig. 5.

"With this instrument I can lay hold of the neck of the tumour more readily than by any other means. Before endeavouring to extirpate the polypi, it will be necessary to ascertain exactly the situation of the origin of the excrescence: and for that purpose a careful examination of the parts will be absolutely necessary. The inspection may be accomplished in the rays of the sun, or by means of the inspector auris, and the auricular probes.

"When the site of the origin of the polypi has been accurately ascertained, and the patient seated as directed in the other operations of the ear, with the head well secured, the operator should then lay hold of the auricle with the left hand, and bring the meatus in a line with the rays of the sun, or those emitted by the inspector auris; and with the forceps in his right hand he should then introduce the instrument cautiously into the tube (with a blade on each side of the tumour) until he has got hold of the cervix.

"He should then grasp the forceps firmly, and give the instrument a half turn, and at the same time pull towards him; and by this means the tumour will in general be torn from its origin and conveyed out of the meatus." 75.

The parts being very vascular, sometimes a considerable quantity of blood is lost after extirpation—a loss more alarming than dangerous—more salutary than injurious. In recent polypi our author has frequently succeeded in destroying the excrescence by touching the tumour slightly with a camel-hair pencil dipped in the tinct. ferri muriat. renewing the application once daily till they were destroyed.

8. *Purulent discharge from the Meatus.* Inflammation of the meatus is too generally neglected by the patient, or improper remedies employed. Hence ulceration is the frequent consequence. Our author makes three species of inflammation of the meatus, viz. Pain *without* diminution of hearing—pain *with* diminished hearing—and pain with diminished hearing and discharge.

"*Symptoms.* The symptoms, in the *first species* are few: namely, tenderness of the parts—uneasy sensations in the meatus, accompanied with a greater or less degree of pain, according to the state of the inflammation.

"In the *second species* the pain is generally more acute than in the first, and accompanied with diminution of hearing—meatus reddish coloured—integuments and cerumenous glands swollen, so as to contract the diameter of the tube, the orifice of which is sometimes almost impervious. The closure of the meatus is caused by the tumefaction of the cerumenous glands and integuments.

"In the *third species* the symptoms are in general not quite so severe as in the first or second; but there is a discharge of purulent matter, in addition to pain and uneasy sensations in the meatus.

"The pulse is in general quicker than usual, accompanied sometimes with rigors and other symptoms of fever, which quickly subside on the formation of matter; but if the patient be costive these symptoms are heightened, with general uneasiness and pain in the parts.

"The cerumenous glands participate in the change, and the secretion is gradually altered into thin, yellowish matter—sometimes of a greenish hue; and if the discharge should continue for a length of time, without any attempt being made to relieve the patient, it soon acquires a fetid, disagreeable smell; erosion of the integuments takes place, and great part of the tube is converted into an ulcerated surface.

"The discharge accumulating and lodging in the interior depression of the tube, sometimes causes a considerable diminution of hearing, especially in patients of a scrophulous diathesis, where the matter is in general thick and curdled, or thin, acrid and mixed with blood." 84.

The causes are various—irritation from extraneous substances—cold, &c.

*Treatment.* In the first species leeches should be clustered round the ear, and the purgative medicines exhibited.

Where suppuration has taken place, and consequently where there is a discharge from the meatus, the ulcerations should be healed as soon as possible, lest the surrounding parts should be irreparably injured. The tube should be washed out four or five times a day, with tepid milk and water; and a bitter aperient saline medicine exhibited. Few recent cases will resist this simple treatment, if carefully employed, and the patient kept confined to his room. But exposure to the air, in this state is liable to produce violent inflammation. If the patient cannot be confined, the tepid injection should be discontinued, and a dilute solution of nitrate of silver (one grain to ℥x. of distilled water) or sulphate of zinc, used. When there is considerable diminution of hearing, as well as purulent discharge, our author gives mercurial preparations, with opium and antimony till the constitution is slightly affected. Our author also experienced very good effects, in many cases, from the use of iodine, and thinks it may often be advantageously substituted for mercury.

Among his dispensary patients, however, Mr. Buchanan was obliged to seek for a remedy "that should cure the discharge in a moderate space of time, and restore the hearing *instantaneously*."

"After a careful investigation of the properties of various medicines, applicable to the treatment of Inflammatio Suppurata; and after watching their effects on patients affected with this disease, I was so fortunate as to find one, which though extensively used for mercantile and chymical purposes, had as yet never been administered for the cure of diseases of this organ.

"In a very obstinate case of purulent discharge from both ears, with considerable diminution of hearing, that had been submitted to a variety of medicines, without any permanent benefit, I was induced to try a weak mixture of Pyrolignous Acid, in the form of an injection, and found it to surpass my most sanguine expectations in diminishing the discharge, and almost *immediately restoring the hearing*.

"In a few days the patient (a female) on whom the experiment was made, was agreeably surprised to find that she could distinguish whatever was spoken to her, though pronounced in the softest whisper. The discharge was entirely divested of its fetid smell, and gradually diminished in quantity, and the patient was relieved from a very disgusting and offensive complaint.

"I have made repeated trials of this injection, and find it succeed in restoring the hearing in every case of purulent discharge, accompanied with diminution of hearing; and I may venture to say that a more valuable medicine than that of Pyrolignous Acid has not been introduced into acoustic surgery either in this or the last century." 97.

Cases in illustration are related by our author, for which we refer to the work itself. The form of the injection is given a few pages back, and it is to be varied in strength, according to circumstances. The mode of applying it is, first to wash out the parts with tepid water, and then to inject the fluid, so as to be directly applied to the abraded or ulcerated surface. The effects produced by this injection are, first giddiness, succeeded in a few minutes by an agreeable warmth—sensation of lightness in the head—and then a restoration of hearing. During the use of this injection the bowels should be kept open.

We have thus given a condensed view of Mr. Buchanan's little work, which we think will prove useful to the professed aurist, as well as to the general practitioner. It is necessary, however, that the plates should be consulted in order that the surgeon may derive the utmost advantage from the operative portion of the work, and the inventive genius of its author.

